# Browning of White Adipose Tissue as a Therapeutic Tool in the Fight against Atherosclerosis

**DOI:** 10.3390/metabo11050319

**Published:** 2021-05-14

**Authors:** Christel L. Roth, Filippo Molica, Brenda R. Kwak

**Affiliations:** Department of Pathology and Immunology, University of Geneva, CH-1211 Geneva, Switzerland; christel.roth@etu.unige.ch (C.L.R.); brenda.kwakchanson@unige.ch (B.R.K.)

**Keywords:** white adipose tissue, brown adipose tissue, adipocytes, browning, non-shivering thermogenesis, β3-adrenergic stimulation, cold exposure, atherosclerosis, atheroprotection

## Abstract

Despite continuous medical advances, atherosclerosis remains the prime cause of mortality worldwide. Emerging findings on brown and beige adipocytes highlighted that these fat cells share the specific ability of non-shivering thermogenesis due to the expression of uncoupling protein 1. Brown fat is established during embryogenesis, and beige cells emerge from white adipose tissue exposed to specific stimuli like cold exposure into a process called browning. The consecutive energy expenditure of both thermogenic adipose tissues has shown therapeutic potential in metabolic disorders like obesity and diabetes. The latest data suggest promising effects on atherosclerosis development as well. Upon cold exposure, mice and humans have a physiological increase in brown adipose tissue activation and browning of white adipocytes is promoted. The use of drugs like β3-adrenergic agonists in murine models induces similar effects. With respect to atheroprotection, thermogenic adipose tissue activation has beneficial outcomes in mice by decreasing plasma triglycerides, total cholesterol and low-density lipoproteins, by increasing high-density lipoproteins, and by inducing secretion of atheroprotective adipokines. Atheroprotective effects involve an unaffected hepatic clearance. Latest clinical data tend to find thinner atherosclerotic lesions in patients with higher brown adipose tissue activity. Strategies for preserving healthy arteries are a major concern for public health.

## 1. Introduction

In the past few years, adipose tissue (AT) understanding evolved from an inert fat storage tissue to a complex organ interacting with the whole body [1]. It plays a leading physiological role in metabolic and energy homeostasis. Conversely, AT dysregulation is implicated in the metabolic syndrome leading to metabolic disorders like obesity, type II diabetes and cardiovascular diseases, including atherosclerosis. Mammals have two distinctive types of AT: white (WAT) and brown (BAT), classified by their cells, colors, locations, precursors, vascularization, innervation and antagonist activities.

WAT, the central body fat mass extensively distributed in several visceral and subcutaneous depots, has two physiological functions [2]. It acts as energy storage; its white adipocytes store droplets of triglycerides in times of plethora and hydrolyze them in times of lacking. WAT is also a specialized organ that secretes several active compounds, adipokines, as auto-, para-, and endocrine signals [3]. In relation to other organs, these adipokines are the main actors in energy homeostasis and immunity, endothelial and blood pressure maintenance [4,5,6,7]. WAT dysregulation is associated with obesity, especially the expansion of visceral AT depots, and is characterized by an altered adipokine secretion. The latter conducts adipocyte hypertrophy and hyperplasia, and modifications in AT’s structural cells, such as vascular and immune cells, lead to the pro-inflammatory state associated with the metabolic syndrome [8].

BAT is a high metabolic tissue with a primary thermogenic function. The high amount of mitochondria and vascularization results in the distinct brown color of BAT [9]. In humans, BAT is located in the cervical, axillar, perirenal, adrenal, mediastinal, paravertebral and upper abdominal regions along large blood vessels, trachea and intercostal arteries [10,11]. Brown adipocytes specifically express uncoupling protein 1 (UCP1), a mitochondrial protein. UCP1 dissipates energy in the form of heat rather than ATP production [12]. Thus, BAT limits WAT storage and decreases hyperlipidemia by increasing energy expenditure [13]. Besides, WAT dysfunction and accumulation reduce BAT activity, the white fat percentage being inversely related to it [14]. The hypothesis of using BAT activity to induce calory burning via fatty acid oxidation was proposed more than a decade ago [15]. Several studies in humans have identified BAT activation as a potential therapeutic target for increasing fat loss [14,16].

**Figure 1:**
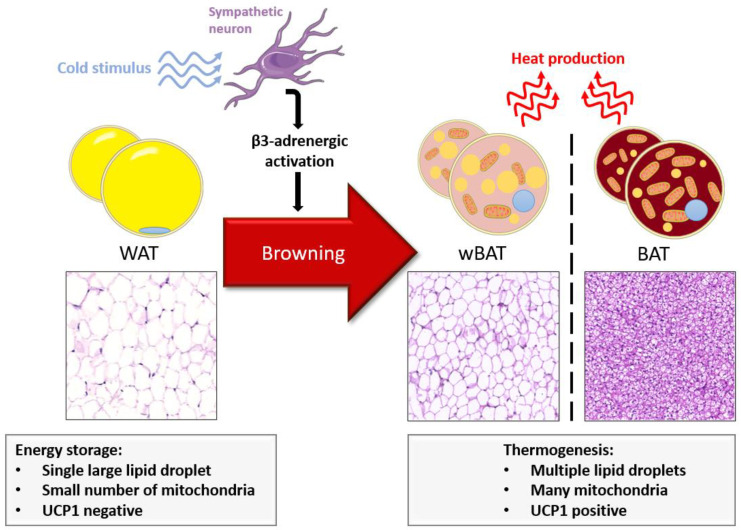
Cold exposure physiologically induces the browning process by sympathetic fiber innervation via a β3-adrenergic stimulation of white adipose tissue (WAT). The beige phenotype, enriched with mitochondria, allows for non-shivering thermogenesis like brown adipocytes due to the expression of UCP1. Photographs represent hematoxylin and eosin (HE) staining of WAT, WAT-derived brown-like adipose tissue (wBAT) and brown adipose tissue (BAT) in mice.

More recently, the induction of UCP1 expression in white adipocytes has been shown in response to various stimuli [17]. The thermogenic cell differentiation in WAT is called browning (Figure 1). WAT-derived brown-like AT (wBAT) has similar characteristics as BAT, like a large number of mitochondria and high metabolic activity. Still, BAT and wBAT are two distinctive tissues with their own morphology, gene expression and embryogenic precursors, suggesting different regulatory signals [17]. β3-adrenergic receptor activation is a main browning stimulation pathway, initially identified by the density of noradrenergic fibers in wBAT [18]. In response to cold exposure, insulin and β3 agonists, increased wBAT and resistance to obesity have been reported in mice [18]. Research in humans reveals promising results for inducing browning of WAT [19,20,21].

In the present review, the potential benefits of BAT activation and WAT-to-BAT trans-differentiation will be discussed in the context of atherosclerosis, focusing on the therapeutic targets.

## 2. Thermogenic Adipose Tissue

Adaptive thermogenesis defines the body temperature regulation in response to cold exposure. The stimuli-induced heat generation derives from shivering-or muscular-and non-shivering forms [22]. The second one depends on the UCP1-related activity of the thermogenic BAT and wBAT. Despite the same heat-producing ability, inherent differences between BAT and wBAT depict them as two distinct AT.

### 2.1. BAT and wBAT: Two Different Adipogenesis

BAT is a thermogenic AT already present at birth. Brown adipocytes arise from a muscle-like cellular lineage, independent of the white adipocyte lineage [23]. They express transitorily *myf5*, previously regarded as a specific skeletal muscle gene, during embryogenesis [24].

wBAT biogenesis is not embryogenic. Its *de novo* formation is generated by the browning of WAT during the whole life in response to environmental stimuli. wBAT is reverted to WAT in their absence [25]. Phenotypically, brown-like WAT-derived adipocytes, called beige cells, are between white adipocytes, with their unique large lipid droplet, and the polygonal shape and multilocular lipid droplets typical for brown adipocytes [26]. The postnatal arising of beige adipocytes remains unresolved [24]. The three prevalent hypotheses for *de novo* formation of beige cells are a stimuli-induced differentiation in WAT from a specific beige precursor, a white preadipocyte, or from a trans-differentiation of a mature white adipocyte [27,28]. Research predominantly supports that beige cells do not originate from a BAT muscle-like lineage precursor despite their common properties [24,26].

### 2.2. Brown and Beige Cells Shared Gene Expression

BAT and wBAT share a similar thermogenic and adipogenic gene expression panel (Table 1) [26,27]. Their expression manages thermogenesis, prenatal development of brown cells but also the induction of WAT browning. To date, this parallel gene expression appears to be key for understanding the emergence of beige adipocytes.

Peroxisome proliferator-activated receptor-gamma (PPAR-γ), the leading adipogenic differentiation factor expressed in brown, beige and white adipocytes, is sufficient to promote adipogenesis [24,29]. Its activation up-regulates adipogenic genes and decreases myogenic gene expression in BAT [24]. Many identified coactivators regulate PPAR-γ transcriptional activity, and the complex of specific coactivators/PPAR-γ binds to several promotors.

PPAR-γ coactivator-1α (PGC1-α) is expressed in tissues with high energy demand and oxidative capacities such as the heart, skeletal muscle, and thermogenic AT. Focusing on BAT and wBAT physiology, PGC1-α is a crucial factor for thermogenesis [30]. Cold exposure and β3-adrenergic signaling induce its expression [30]. The complex PGC1-α/PPAR-γ binds the promotor of the thermogenic gene *Ucp1* [30]. Forkhead Box C2 (FOXC2), another transcription factor, is linked to BAT embryogenesis and WAT browning. PGC1-α and FOXC2 both play a key role in the mitochondrial biogenesis [31,32,33]. PGC1-α and FOXC2 are not expressed in mature white adipocytes and their induction triggers WAT browning [30,34,35].

PPAR-γ is also co-activated by the expression of PR domain containing 16 (PRDM16), a transcription factor essential for BAT development that controls the switch of muscle-like precursors into brown adipocytes by activating specific adipogenic genes like *Ucp1* [24,36]. Besides, PRDM16 is also involved in the β3-adrenergic-dependent recruitment of beige adipocytes in subcutaneous WAT [37]. PRMD16 is primordial for beige adipocyte maintenance and browning induction [26]. Lack of PRDM16 is associated with WAT reconversion from wBAT [26]. PRDM16 expression arises from the increase of FOXC2 [26,32].

The potential of PPAR-γ-dependent adipocyte proliferation is well regulated. In-vitro studies reveal that the retinoblastoma protein (pRb) synthesized from RB1, a tumor suppressor, physiologically represses PPAR-γ expression, decreasing BAT formation [23,38]. pRb absence in mouse models promotes BAT prenatal adipogenesis, whereas *RB1* double allele loss leads to hibernomas, BAT tumors [38]. Moreover, pRb is involved in the early stages of beige adipocytes differentiation and its downregulation stimulates browning [23,38]. pRB indirectly impairs browning by repressing PGC1-α and FOXC2 expression [35].

UCP1, PPAR-γ, PGC1-α, FOXC2, PRDM16 and pRb appear to so far have the main implication for the induction and maintenance of brown and beige cells [26,39]. Nonetheless, other proteins also seem relevant for adipogenesis and thermogenesis.

PPAR-α, another protein from the PPAR family, is also present in thermogenic AT. It shares some coactivators with PPAR-γ, such as PGC-1α or PRDM16. Its transcriptional activity is another essential pathway for UCP1 expression [40].

Interferon regulatory factor 4 (IRF4) regulates adipogenesis via the induction of *Pparg* and *Prdm16* expression and its interaction with PGC-1α. The latter is unable to induce *Ucp1* expression without IRF4 [39]. Lack of IRF4 in mice is associated with a decrease of UCP1 and PGC1-α in their BAT and leads to obesity, insulin resistance, and an intolerance to cold [41].

Fibroblast growth factor 21 (FGF21) is a protein highly implicated in metabolic regulation. The liver and the AT secrete it in endocrine, paracrine and autocrine ways. *Fgf21* is a crucial gene in BAT activation and browning of WAT [42,43]. It stimulates PPAR-γ activity and PGC1-α expression, and enhances UCP1 production, increasing the thermogenic activity in beige and brown cells [42,44,45].

Nuclear Factor I A (NFIA) is a transcription factor working in parallel with PRDM16 and co-activates PPAR-γ leading to the cascade of embryogenic formation of BAT and recruitment of wBAT [36].

Cell death-inducing DNA fragmentation factor-like effector A (CIDEA) is a lipid-droplet coupled protein acting as a transcriptional regulator [46]. It is endogenously expressed in humans and CIDEA in BAT regulates UCP1 by suppressing its transcription, decreasing thermogenesis [46,47]. Conversely, CIDEA in wBAT up-regulates the expression of UCP1 and is necessary to induce browning [46].

CCAAT/enhancer-binding protein (CEBP) is a member of another family of transcriptional regulators implicated in adipocyte differentiation. This transcription factor acts early in the adipogenic activation cascade, being transitorily expressed in preadipocytes. It induces PPAR-γ expression, resulting in adipogenesis and a thermogenic AT phenotype [48]. CEBP and PPAR-γ are closely related due to a reciprocal expression of their gene [48,49].

ELOVL Fatty Acid Elongase 6 (ELOVL6) and ELOVL Fatty Acid Elongase 3 (ELOVL3) bind fatty acids in long chains. ENVOL6 is necessary for BAT thermogenic activity and regulates the mitochondrial function [50]. Its increase was demonstrated in the cold-exposed mice in correlation with BAT thermogenic activity. ELOVL3 appears to have an important role in lipid recruitment during cold exposure.

Finally, prolactin hormone (PRL) and its receptor PRLR are essential for embryogenic BAT establishment by increasing PPAR-γ expression. Furthermore, it controls wBAT differentiation through pRb signaling [27]. The lack of PRL in mice leads to BAT hypotrophy, whereas the lack of PRLR is associated with an absence of brown adipocyte differentiation [27].

**Table 1:** Overview of common genes expressed in both brown adipose tissue (BAT) and white adipose tissue-derived brown-like adipose tissue (wBAT) with adipogenesis and/or non-shivering thermogenesis implications. They constitute potential targets for induction of browning and BAT activity.

Adipogenic and Thermogenic Genes Expressed in BAT/wBAT	Related Protein	Key-Roles in BAT/wBAT Function	Ref.
*Ucp1*	UCP1	Mitochondrial protein	[12,40]
		Non-shivering thermogenesis	
*Ppar-γ*	PPAR-γ	Nuclear receptor protein	[24,25,29]
		Transcription factor	
		Master role in adipogenesis	
*Ppar-α*	PPAR-α	Nuclear receptor protein	[40]
		Transcription factor	
*PGC1-α*	PGC1-α	PPAR-γ and PPAR-α coactivator	[30,31,35]
		Mitochondria biogenesis	
*Foxc2*	FOXC2	Transcription factor	[32,33,35]
		Mitochondria biogenesis	
*IRF4*	IRF4	Transcription factor for *Ppar-γ* and *Prdm16*	[39,41]
		Transcription of *Ucp1* with PGC1-α interaction	
*Prdm16*	PRDM16	Transcription factor	[24,26,32]
		PPAR-γ and PPAR-α coactivator	
		In wBAT: beige adipocyte differentiation and maintenance	
*RB1*	pRb	Tumor suppressor protein	[23,35,38]
		Inhibitor of PPAR-γ expression and adipogenesis	
		*RB1* downregulation is associated with BAT/wBAT differentiation	
*Fgf21*	FGF21	Metabolic regulator	[42,43,44,45]
		Promotes PPAR-γ activity and PGC1-α transcription	
*NFIA*	NFIA	Transcription factor	[36]
		PPAR-γ coactivator	
*CEBP*	CEBP	Transcription factor of PPAR-γ	[48,49]
		Role in adipogenesis and brown/beige phenotype	
*Elovl6*	ELOVL6	Fatty acid elongase	[50]
		Indirect thermogenesis regulator	
*Elovl3*	ELOVL3	Fatty acid elongase	[50]
		Implication in lipid recruitment	
*CIDEA*	CIDEA	Transcription regulator	[46,47]
		In BAT: decreases UCP1 expression	
		In wBAT: implicated in the browning process and increases UCP1 transcription	
*PRLR*	PRLR	Prolactin receptor	[27]
		Activation increases PPAR-γ expression	
		In BAT: embryogenic adipogenesis	
		In wBAT: browning induction	
*ADRB3*	β3-adrenergic receptor	Noradrenergic activation pathway	[22,33,40,51,52,53]
*DIO2*	DIO2	Thyroid hormone activation pathway	[54,55]

### 2.3. BAT and wBAT in Pathophysiological Conditions

Another common link between BAT and wBAT is their shared activation pathways. Thermogenic AT physiological activity is directly related to the environmental temperature and internal metabolic activity. According to the similar stimuli-induced recruitment, both thermogenic AT operate in parallel [56]. The activation mechanisms are various.

The central nervous system carries out the control of thermogenesis of BAT and wBAT. Several stimuli, such as cold exposure, pre- or postprandial state, endocannabinoids, and melanocortin, are integrated into the brain centers located in the hypothalamus, involved in temperature and energy homeostasis [57]. The preoptic area is a hypothalamic nucleus identified as the main thermoregulator center. It receives signals from cold-activated skin thermoreceptors [57]. Stimulation of the preoptic area regulates other hypothalamic nuclei involved in thermoregulation, i.e., dorsomedial hypothalamus, paraventricular hypothalamus, ventromedial hypothalamus, lateral hypothalamus and arcuate nucleus [57]. They send efferent outputs via the sympathetic nervous system fibers, which heavily innervate the whole AT [52,57]. The postganglionic nerves release noradrenaline, activating the β3-adrenergic receptor (Table 1) on the cell membrane of white, beige and brown adipocytes [57,58]. Thus, the β3-adrenoreceptor has the master role known in non-shivering thermogenesis [52]. It is coupled with a protein G, which activates adenylate cyclase, increasing cyclic adenosine monophosphate (cAMP) and leading to protein kinase a (PKA) activation [59]. PKA, associated with the cAMP responsive element binding protein (CREB), regulates gene expression via its phosphorylation ability [59]. The downstream activation cascade leads to the induction of PGC1-α and PPAR-γ, mitochondria biogenesis, PRDM16 expression, and finally results in the increase of UCP1-related energy dissipation and heat production [22,26,33,40,52]. Besides, it induces lipolysis, releasing free fatty acids as energetic substrates for conversion into heat [57]. Noradrenergic stimulation of WAT leads to browning [52,53]. Cold exposure activates the noradrenergic receptor pathway. This BAT and wBAT cold-stimulated pathway has already been demonstrated in rodents and humans [22,51,52,60].

The type 2 iodothyronine deiodinase (DIO2) expressed in BAT and wBAT is another protein involved in adaptive thermogenesis (Table 1). It is independent of the adrenergic pathway. It activates thyroid hormones, promoting thermogenic gene expression and metabolism by their direct action in increasing cellular transcription [54,55].

BAT and wBAT dysregulation appears to be relevant in pathophysiological conditions due to their central role in energy homeostasis. Weight shows an inverse correlation with BAT metabolism. Indeed, individuals with obesity have a decreased adrenergic-dependent thermogenic activity [22,61]. In thyroid disorders, thermogenic and weight effects occur due to thyroid hormone AT-related function. Hypothyroidism leads to hypothermia and weight gain. Opposite effects occur in hyperthyroidism [54].

### 2.4. Research on AT Browning

The potential of the thermogenic cell differentiation in AT seems a promising therapeutic tool. Research in mice and humans reports browning induction pathways.

Cold exposure seems the most physiological therapeutic tool, using its adaptive purpose [62]. It has shown promising effects in mice and humans [60,63,64]. Positive results in mice for induction of browning have already been demonstrated with β3-adrenergic receptor agonists and PPAR-γ agonists [25]. However, several assays with adrenergic agonists were unsuccessful in humans due to lower specificity of the receptor and oral availability [15]. Thiazolidinediones, a group of drugs used in diabetes to increase insulin sensitivity, are synthetic agonists of PPAR-γ [65,66]. In mice, they lead to the subsequent PRDM16 expression and activate the cascade to AT browning [17,46]. A similar effect has yet to be established in humans.

Cyclooxygenase 2 (COX-2), a synthesizing enzyme prostaglandin, forms a downstream part of the β3-adrenergic activation cascade in WAT and is necessary for wBAT production. The overexpression of COX-2 induced by cold exposure or by β3-adrenoreceptor agonists was observed in WAT depots, in association with wBAT phenotype. Furthermore, the induction of browning is decreased in COX-2 knock-out mice [67]. Importantly, COX-2 inhibition reduced the weight loss in cachectic cancer patients [68]. In this study, the decrease in resting energy expenditure may be partly due to an effect on UCP1-related AT [67,68]. However, the anti-inflammatory and increased appetite effects of COX-2 inhibitors complexify understanding of their mechanisms in humans [67].

Many other activation pathways are currently under investigation. Due to its significant role in adaptive thermogenesis and wBAT induction, PGC1-α constitutes an optimal target. Two PGC1-α activation pathways are relevant, the upstream stimulation of estrogen-related receptor α and the activation of the silent information regulator/sirtuin-1 [33]. Both stimulated the increase of functional PGC1-α. Recent studies have revealed the implication of the PRL pathway in the induction of browning. PRLR knock-out and an absence of PRL signaling render mice resistant to obesity due to the high emergence of wBAT [27]. Additional activation pathways are also under investigation, like natriuretic peptide, insulin, or irisin, a newly discovered hormone released by myocytes during physical activity [17].

Emerging evidence points to physical exercise training stimulating the browning of WAT via secreted myokines and hepatokines [69,70,71,72], an effect that is mainly observed in lean rodents [69,73]. Conflicting results have been reported however in obese rodents in which exercise training may induce increases as well as decreases in typical browning markers [74,75]. Moreover, the housing temperature seems to influence the ability of physical exercise to increase markers of mitochondrial biogenesis and the browning of WAT [76].

wBAT has a unique gene expression profile, with transcription factors, metabolism-related and inflammatory-associated proteins that are not present in BAT. Nevertheless, roles of specific wBAT protein remain unclear, and the difference between brown and beige adipocyte physiology is still poorly understood [53,77]. Recent studies reveal that beige cells also have an UCP1-independent thermogenic activity, in contrast to the exclusively UCP1-dependent thermogenesis in BAT [25]. Although UCP1 is still considered as constitutive of non-shivering thermogenesis, creatine substrate cycling and Ca^2+^ cycling may change this conception [25]. Although not yet completely understood, their mechanisms appear distinct from UCP1 and show specificity for wBAT. These data suggest that *de novo* formation of beige cells may significantly influence the whole non-shivering energy expenditure in humans [14,16].

Mouse models are useful to understand the details of BAT and wBAT physiology and may be helpful in the discovery of therapeutic targets. Nevertheless, their translation to human therapy will remain challenging. To date, cold exposure seems the safest way to induce wBAT differentiation and BAT activity. Therefore, clinical research about BAT activity and browning, notably in atherosclerosis, is based on this application.

## 3. Atherosclerosis

### 3.1. Pathological Process

**Figure 2:**
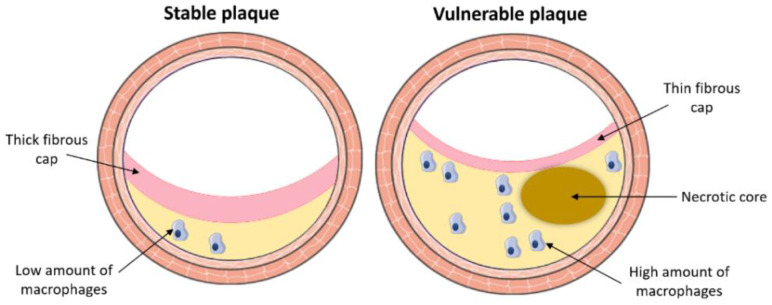
Atherosclerotic plaque vulnerability, an important factor for acute cardiovascular events, is due to lipid deposition, macrophage invasion and necrotic core formation. On the contrary, the fibrous cap, composed of smooth muscle cells and collagen, stabilizes the atherosclerotic plaque and protects it against rupture.

Atherosclerosis, the number one cause of death and disability in Western societies, is a chronic lipid-driven inflammatory disease characterized by the formation of atheromatous plaques in walls of medium and large-size arteries. Atherosclerotic plaques develop in arterial sections exposed to disturbed blood flow, inducing endothelial dysfunction [78,79]. Endothelial dysfunction leads to sub-endothelial trapping of low-density lipoprotein (LDL) in the intima. Consecutive inflammatory response and LDL oxidation further activate the endothelial cells with the induction of adhesion molecule expression. Monocytes recruited into the lesion phagocyte oxidized LDL, becoming highly pro-inflammatory macrophage foam cells. Activated smooth muscle cells migrate from the media to the intima. Increased macrophage cellular stress leads to macrophage necrosis and necrotic core formation [80]. The end result is an atheroma, composed of inflammatory cells, mainly macrophages participating in lipid clearance, a necrotic core and activated smooth muscle cells secreting extracellular matrix, mostly collagen, which forms a fibrous cap that covers and protects the atheroma from rupture (Figure 2).

Rupture of an atherosclerotic plaque may lead to acute cardiovascular events, such as myocardial infarction or stroke. The disrupted endothelium exposes the pro-thrombotic content of the atherosclerotic plaque interior to the blood, and the formation of the subsequent occlusive thrombus blocks the blood flow, with destructive consequences for the downstream tissue. The growth of atherosclerotic plaques can also cause chronic cardiovascular disorders because of arterial stenosis and blood flow reduction.

Two factors are associated with pathological outcomes: the size and the stability phenotype of atherosclerotic plaques. Plaque vulnerability is mainly due to lipid deposition and macrophage invasion as well as necrotic core formation. On the contrary, the fibrous cap, composed of smooth muscle cells and collagen, increases the stability of atherosclerotic lesions (Figure 2) [81]. Cardiovascular risk factors affect both growth and vulnerability. The main cardiovascular risk factors related to metabolic syndrome are visceral obesity, hypertriglyceridemia/high LDL level, low high-density lipoprotein (HDL) level, hypertension, and high blood glucose level [82,83].

### 3.2. WAT Worsens Atherosclerosis

**Figure 3:**
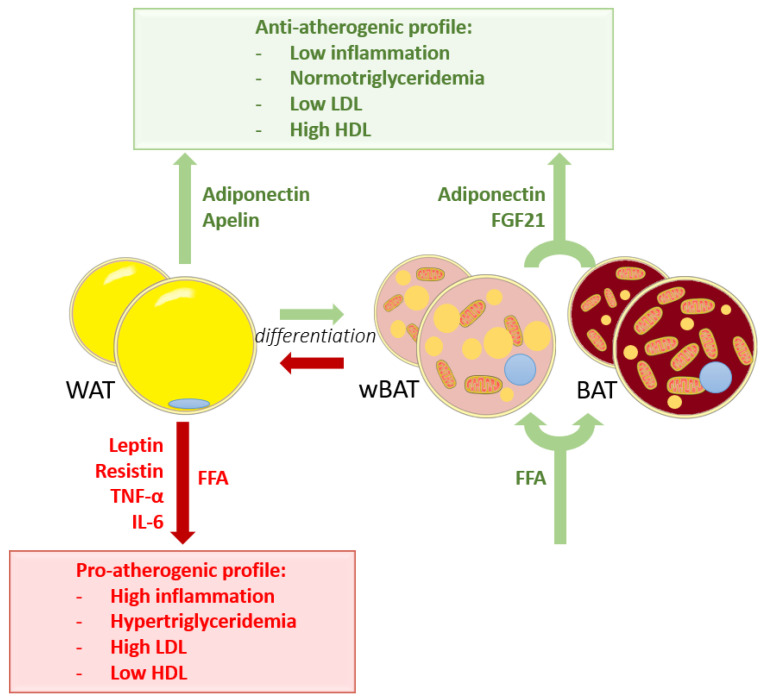
Schematic representation of the relation between adipose tissue and atherosclerosis. Under physiological conditions, brown adipose tissue (BAT) and white adipose tissue-derived brown-like adipose tissue (wBAT) activation leads to an atheroprotective profile via the consumption of free fatty acids (FFA) and the secretion of adiponectin and FGF21. Subcutaneous white adipose tissue (WAT) increases the atheroprotection by the secretion of adiponectin and apelin. In WAT dysregulation, FFA and pro-atherogenic factors are released, increasing the development of atherosclerosis and augmenting plaque vulnerability.

WAT dysfunction in the obesity state seems to play a decisive role in the development of atherosclerosis and plaque stability. Some mechanisms give clues for a principally unknown interaction (Figure 3).

Increased lipolysis in WAT ensures a catabolic state [84]. Higher adrenoreceptor sensitiveness in the visceral WAT than subcutaneous WAT is responsible for the sympathetically-induced lipolysis and protects from weight gain [85]. Insulin physiologically inhibits lipolysis in WAT, to permit postprandial lipogenesis [84]. In obesity, the excess of calories engenders WAT hypertrophy, and the oversupply of lipids cannot be stored. It results in ectopic pro-inflammatory fat depots, especially visceral ones [84]. Consequently, these fatty acid secretions lead to the elevated formation of triglyceride-rich lipoproteins by the liver and hypertriglyceridemia, a significant risk factor in atherogenesis [84].

Besides, adipokine secretion by dysregulated WAT is relevantly changed. Perivascular AT (PVAT) surrounds blood vessels. This particular type of AT has WAT and BAT characteristics depending on its location [86]. Coronary artery PVAT morphology is similar to visceral fat and it releases the same adipokines with a local effect. One hypothesis is that adipokines pass from PVAT into the vascular wall via *vasa vasorum* [86,87]. The secretion of adipokines from subcutaneous and visceral WAT and PVAT act on atherosclerotic plaque development and have effects on the vascular wall integrity [88].

Leptin, known for appetite regulation, is a pro-inflammatory cytokine that can promote endothelial dysfunction, growth and vulnerability of atherosclerotic plaques [88,89,90,91,92]. It induces oxidative stress in the atheroma and an up-expression of inflammatory signals [88,90,91]. Leptin level in humans was associated with increased atherosclerotic plaque burden and severity [92,93]. Resistin is a pro-inflammatory adipokine activating endothelial dysfunction and invasion of monocytes into the arterial wall invasion by up-regulating the expression of adhesion molecules in endothelial cells [94,95]. In patients, its plasmatic levels are directly associated with atherosclerosis burden [95]. Adiponectin is a major anti-inflammatory adipokine that inhibits atherosclerotic plaque growth due to a diminution of endothelial adhesion molecules, of oxidized LDL uptake by macrophages, of foam cells formation, and of smooth muscle cells differentiation and migration [92,96,97,98]. A low level of adiponectin is a good predictor for risk of myocardial infarction in patients [92]. Apelin is a peptide implicated in smooth muscle cell integrity and inhibits foam cell formation [84,99,100]. Its plasmatic levels are low in patients with acute myocardial infarction [100]. In the obesity state, pro-atherogenic leptin and resistin are over-secreted, whereas anti-atherogenic adiponectin and apelin are under-expressed. Alteration in adipokines leads to a pro-atherogenic imbalance.

In addition to other tissues, WAT may also release tumor necrosis factor alpha (TNF-α) and interleukin 6 (IL-6), two central pro-inflammatory cytokines. They induce systemic inflammation and, specifically in atherosclerosis, internal plaque inflammation and enhance plaque vulnerability [4]. They are both elevated during infection and inflammation and have an extensive systemic response. For instance, TNF-α is involved in the induction of fever and increases other acute-phase proteins, like IL-6 and C-reactive protein (CRP). Several direct effects in atherosclerotic plaque growth are known, like induction of endothelial adhesion molecule expression [101]. IL-6 is an essential activator of the immune system. Pro-inflammatory states, like obesity, are associated with elevated plasmatic level of IL-6 and show a correlation with atherogenesis and acute cardiovascular events [102]. Although the correlation between levels of TNF-α and IL-6 and atherosclerosis are well established, their direct influence on progression of the disease is not precisely established due to their vast systemic pro-inflammatory effects [101,102].

In humans, several studies have shown a correlation between the visceral WAT volume and the amount of atherosclerosis [103]. In particular, the visceral fat deposit correlates with well-established cardiovascular risk markers: hypertension, insulin resistance, hypertriglyceridemia and pro-atherogenic cholesterol profile [104].

### 3.3. Influence of BAT on Atherosclerosis

The interest in the influence of BAT on atherosclerosis has emerged only recently and appears to be a potential target to reduce atherosclerotic plaque expansion (Figure 3). BAT possibly has both systemic and local effects on atherogenesis.

Using different mouse models, research has highlighted that BAT plays a systemic anti-atherogenic role. Studies of Bartelt et al. in cold-exposed mice reveal that BAT activation decreased hyperlipidemia, notably by reducing LDL plasmatic level [13]. This correlated with increased triglyceride and free fatty acid consumption in BAT (Figure 3) with the associated transcriptional elevation of lipoprotein lipase, an enzyme for lipid degradation, and CD36, a receptor for lipid uptake in BAT. The increased lipid consumption was correlated to thermogenesis-related activity, shown by the increased expression of *PGC1-α*, *Ucp1* and *DIO2* genes. At the same time, HDL plasmatic level increased, improving the cardiovascular profile conjointly with the reduced LDL level [13]. In 2015, Berbée et al. directly connected BAT activation via a β3-adrenoreceptor agonist stimulation in hyperlipidemic mice, with an attenuation of atherosclerosis development. They also noticed a similar effect in LDL reduction and, after ten weeks of treatment, recorded an associated attenuation in the severity and growth of atherosclerotic lesions by about 43% [105].

A surprising study from Dong et al. about cold-activated BAT described an increase of atherosclerosis development and enhancement of plaque vulnerability in Apolipoprotein E (*Apoe*^−/−^) and LDL receptor (*Ldlr*^−/−^) knock-out mice, well-known mouse models for the disease. However, the same cold condition was beneficial for the lipidic profile of wild-type mice and decreased atherogenesis [106]. Another recent study reported increased atherosclerosis development in *Apoe*^−/−^ and *Ldlr*^−/−^ mice treated with a β3-adrenoceptor agonist [107]. Both the *Apoe*^−/−^ and *Ldlr*^−/−^ mice showed reduced clearance of triglycerides-rich apolipoprotein remnants by the liver. In this line, the anti-atherogenic effect of BAT activation was shown to depend on the conserved hepatic lipid clearance [105].

CEPT mice are a well-established model for human-like lipoprotein metabolism and atherosclerosis, with no modification remnants clearance by the liver [105]. CEPT mice have a higher HDL concentration after β3-adrenergic stimulation by cold exposure or by β3-adrenergic agonists [108]. Thermogenic AT modulates the HDL level by the activation of the lipoprotein lipase. HDL reverse cholesterol efflux is increased, boosting conversion of cholesterol in bile acids [108].

It has been recently discovered that BAT secretes many factors, although their functions are not well-established [109]. FGF21 and adiponectin are two identified molecules with an atheroprotective behavior released by activated BAT (Figure 3), and their potential systemic and local anti-atherogenic effects have been suggested [110]. FGF21, in addition to its thermogenic implication in beige and brown cells, has several metabolic functions [42,43]. It stimulates PPAR-γ activity and adiponectin expression, increases lipid and glucose uptake and improves lipid profile by suppressing the nuclear sterol regulatory element binding protein-1, a hepatic factor involved in the biosynthesis of cholesterol [111]; in particular it leads to a decrease of triglyceride and LDL levels and an increase of HDL levels [97,111]. Moreover, FGF21 reduces oxidative stress and inflammation [97]. Its plasmatic levels are associated with thermogenic AT activity and are up-regulated during cold exposure [43,45]. Due to their promising atheroprotective potential, investigations onto adiponectin and FGF21 as therapeutics is underway [97,98]. In humans, several data support that BAT activation may have a global anti-atherogenic effect. A postmortem study showed a significant negative correlation between the BAT volume and coronary atherosclerotic plaques [112]. It appeared that the amount of BAT is also negatively correlated to the visceral WAT volume, measured by waist circumference. In 1998, it was shown that hypercholesterolemic patients receiving twenty minutes of cold exposure per day for three months decreased their LDL and total cholesterol levels [64].

^18^F-FDG PET/CT—an imaging technique using glucose marked by radioactive fluorine—is performed to measure BAT activity making use of its high metabolism [51]. Adopting this method, a study in 443 volunteers negatively correlated BAT activity with arterial inflammation and cardiovascular events [113]. A five-year follow-up study realized by Raiko et al. revealed several borderline correlations between BAT activity, cardiovascular risk markers and atherosclerosis [114]. At baseline, the measurement of BAT activity was performed during cold exposure, insulinemic perfusion, and room temperature. At baseline and five-year follow-up, cardiovascular risk markers were measured and endothelial dysfunction, arterial stiffness and atherosclerosis was estimated with non-invasive ultrasonography techniques, such as flow mediated-dilatation (FMD), pulse wave velocity (PWV) and intima-media-thickness (IMT) [115]. Raiko et al. revealed that individuals with higher BAT activity tended to have smaller atherosclerotic plaques. Cold-activated BAT negatively correlated with triglyceride levels. BAT insulinemic perfusion had a significant correlation with preserved endothelial function. Furthermore, depending on its stimulation, BAT activity tended to be associated with less insulin resistance, lower blood pressure, and higher plasma HDL. For an undetermined reason, correlations were more suggestive in volunteers with average weight than in overweight individuals. Interestingly, the study suggests that the correlation found between supraclavicular BAT activity and brachial endothelial function may result from their nearby location and is a BAT local anti-atherogenic effect. This study puts the idea forward that BAT may serve as a new marker to evaluate subclinical atherosclerosis [114].

### 3.4. Influence of wBAT on Atherosclerosis

In addition to BAT activation, cold-exposed mice have shown increased uptake of triglycerides and fatty acids (Figure 3) by subcutaneous WAT, suggesting that induced wBAT displays a simultaneous role with BAT in the plasmatic triglyceride clearance [13]. A similar WAT browning was observed with β3-adrenoreceptor agonists and was correlated with BAT activation and attenuation of atherosclerosis development [105].

The role of the browning process as a specific activator of anti-atherogenic effects remains unclear. The impact of differentiated wBAT and BAT is complicated because of the similar pathways that play a role in BAT activation and the induction of browning, and the lack of specific genetic tools [56]. Few methods were tried, like adiponectin-specific PRDM16 knock-out mice leading to wBAT deficiency or unilateral denervation of BAT [56,116]. They seem to point to the importance of wBAT activity and metabolic impact, but such approaches have never been combined with atherosclerosis [56]. In human research, the understanding of the individual roles of wBAT seems for now impossible without the global knowledge of the wBAT-BAT shared activity. Investigations still have to be prospected to determine the proportionate influence of either wBAT or BAT in atherosclerosis.

## 4. Discussion and Conclusions

BAT activation and AT browning induction have both already shown their therapeutic potential in metabolic disorders, like obesity and diabetes [56]. In the context of atherosclerosis, some studies already permit an initial view of the potential benefit of thermogenic AT.

### 4.1. Experimental Studies

Data in mice suggest that the combined action of BAT and wBAT tends to decrease the progression and severity of atherosclerotic lesions. BAT activation and WAT browning share the same thyroid hormones and adrenergic stimulatory pathways [20,54,55].

Cold exposure and β3-adrenoreceptor agonists are the predominant stimuli used in mouse models to study thermogenic adipocytes. BAT and wBAT have a high metabolism and convert calories into heat by increasing non-shivering thermogenesis. The induction of browning in WAT activates PRDM16 and FOXC2, essential transcription factors for wBAT differentiation [26,117]. Adrenergic stimulation triggers thermogenesis in BAT and wBAT, driving an intracellular cascade; PGC1-α and PPAR-γ are transcribed, which is essential for the subsequent expression of UCP1 [22,33,40]. Mice that received WAT browning/BAT activation stimuli turn on triglyceride-rich lipoprotein hydrolysis via the lipoprotein lipase, realizing free fatty acids. Some are directly used by beige and brown adipocytes in the β-oxidation for the UCP1-related heat production. The others are released in the blood circulation, involving an enhanced Apolipoprotein E-dependent hepatic clearance of lipid remnants [105]. Activated thermogenic AT enhances the cholesterol conversion in bile acids in the liver, decreasing the total cholesterol and indirectly limiting progression of atherosclerosis [118]. Physical exercise training may be as an additional therapeutic tool. Interestingly, exercise displays important benefits on tissue metabolism, including the one of BAT, and is also implicated in browning of WAT. Investigations conducted in mice and humans report a decreased BAT activity, reduced glucose uptake and lipid storage [62]. However, compared to cold exposure, studies on BAT metabolism and physical exercise training remain highly controversial.

**Figure 4:**
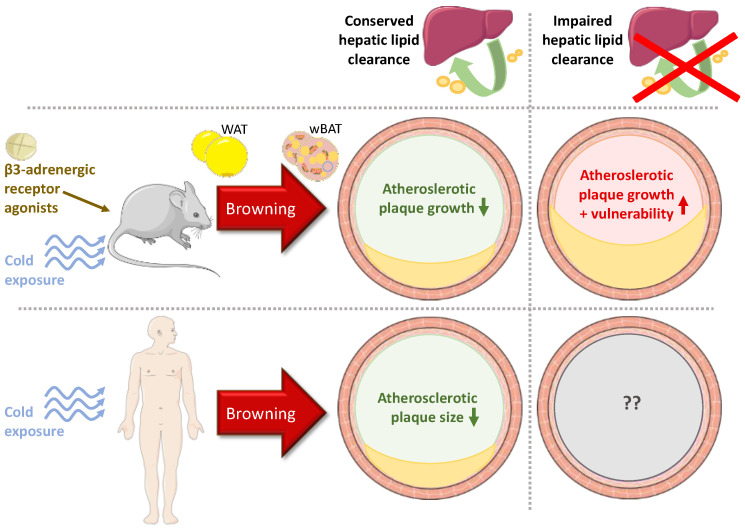
Summary of existing data from preclinical and clinical studies on the effects of white adipose tissue (WAT) browning on atherosclerosis. In the murine model with preserved hepatic clearance of lipids, the stimulation of WAT browning promoted by cold exposure or the use of β3-adrenergic receptor agonists attenuates the development of atherosclerotic plaques. Conversely, the growth of atherosclerotic lesions and plaque vulnerability is promoted in mice with impaired hepatic clearance. In clinical studies, individuals exposed to cold conditions have increased white adipose tissue-derived brown-like adipose tissue (wBAT) differentiation and have smaller atherosclerotic plaques than individuals not exposed to cold. The situation with perturbed hepatic clearance of lipid remnants in atherosclerosis has not been studied in humans.

All these chain reactions in mice improve the cardiovascular risk markers and lead to an anti-atherogenic phenotype (Figure 4). Indeed, mice show decreased plasmatic LDL and triglyceride levels and a rise in HDL levels [13,105]. Besides, BAT has a newly found secretory function. It releases a vast number of molecules, like adiponectin and FGF21, of which the exact effects on atherosclerosis are at present unknown [110].

Browning may be a therapeutic tool to attenuate the development of new atherosclerotic lesions, but it may not act on pre-existing advanced plaques. First, signs of atherosclerotic plaque vulnerability, i.e., high level of inflammatory cells, large necrotic core and small amounts of smooth muscle cells and collagen, seem not to improve by thermogenic AT, while such beneficial effects have been observed for other therapeutics such as statins [119]. Moreover, the thermogenic AT may not stimulate atherosclerotic plaque regression, or possibly it requires an intact hepatic clearance, which reinforces the need for further investigating this topic [119].

Finally, individuals with familial hypercholesteremia with mutations in the *Ldlr* gene have a similar phenotype as *Ldlr*^−/−^ mice, leading to a rapid atheroma accumulation [120]. Like in *Ldlr*^−/−^ mice, familial hypercholesteremia leads to a perturbed hepatic clearance of lipid remnants. Its integrity is essential to procure anti-atherogenic effects of thermogenic AT [13]. This suggests that BAT and wBAT stimulation by cold exposure or β3-adrenoreceptor agonists may lead to the same pro-atherosclerotic impact seen in previous studies conducted in the similar mouse model [105,106,107]. Thus, the browning process is a potential and non-negligible risk factor for the 1 in 250 people with this disease [120].

### 4.2. Clinical Studies

For now, there are very few clinical studies that correlate induction of AT browning and atherosclerosis (Figure 4). However, they indicate a potential atheroprotective role of thermogenic AT activity.

The negative correlation between the amount of BAT and atherosclerotic lesion size suggests anti-atherogenic mechanisms, potentially similar that those found in mice [112]. Data in humans reveal an increase in fatty acid uptake in thermogenic AT—preponderantly in supraclavicular BAT—during acute cold exposure [121,122]. Nevertheless, it accounted for only 0.25% of plasma fatty acid turnover, and unlike the mouse models, plasma triglycerides were not reduced [121]. The increase of free fatty acid production from WAT lipolysis correlates proportionally with thermogenic AT activity [123]. Furthermore, it may be interesting to provide chronic cold exposure to assess a more extensive beige cell differentiation for evaluating potential benefit in plasma lipids.

Chechi et al. have highlighted that the expression of thermogenesis-related genes in *Ucp1*-expressing AT correlates positively with HDL plasmatic level and negatively with the triglyceride level [124]. As previously seen in mice, it seems that BAT and wBAT activity also regulates human triglyceride-rich lipoprotein clearance and directly influences circulating plasma lipids [13,124]. Recently, Raiko et al. revealed a correlation between higher BAT activity, lower plasmatic triglyceride level and smaller in size atherosclerotic plaques [114].

Thus, browning of WAT and thermogenic AT activity may lead to an atheroprotective lipidic profile in mice as well as in humans. An improvement of cardiovascular risk markers, mainly triglyceridemia, may potentially lead to stabilization of atherosclerotic plaques or even to plaque regression [125,126]. However, one should keep in mind that important differences exist between human and rodents with respect to thermogenic capacity and WAT browning which may limit the translation of preclinical studies on mice into clinical practice. Indeed, the stimuli inducing WAT browning in mice have not been systematically investigated for having the same benefits in humans. Furthermore, there exist physiological differences between mice and humans in the location of their fat depots. There is thus an urgent need for more clinical investigations to correlate the improvement of plasmatic lipid levels, atherosclerotic plaque stability, and BAT and wBAT metabolic activity.

### 4.3. Browning vs. Established Atheroprotective Therapies

The supposed WAT browning benefit in atherosclerosis has to be compared with the actual therapeutic methods to establish its usefulness.

Primary prevention against atherosclerosis involves reducing cardiovascular risk factors: smoking cessation, physical exercise, alimentation. These behavioral changes are atheroprotective via metabolic aspects, mutually interconnected. For example, physical activity simultaneously decreases inflammation, oxidative stress, blood pressure, plasma lipids, and increases endothelial function, and muscle energy expenditure [127]. These cumulative effects are a veritable challenge to compare their benefits with WAT browning.

Statins are the most known lipid-lowering drugs and display pleiotropic effects [119,128]. Their main function is to decrease LDL plasmatic level via the inhibition of HMG-CoA reductase, an enzyme suppressing cholesterol degradation. Moreover, statins have other atheroprotective actions such as anti-inflammatory and anti-thrombogenic functions, and endothelial cell preservation. Statin treatment leads to reduced atherosclerotic plaque development but also to plaque regression and reduced plaque vulnerability, two major outcomes that have not (yet) been demonstrated after BAT and wBAT stimulation [119,126,128]. The anti-atherogenic repercussions in well-established plaques recommend using statins in secondary prevention or for primary prevention in severe cardiovascular risk conditions. They reduce the cardiovascular outcomes by one-third, and even if side effects are frequent (e.g., myalgia, headache, nausea), severe reactions remain unusual [129]. Since 2015, an additional class of therapeutics against hypercholesterolemia, i.e., proprotein convertase subtilisin/kexin type 9 (PCSK9) inhibitors, are also used in clinical care. PCSK9 is a hepatic protease that binds the LDLR and stimulate its intracellular degradation via its transport into lysosomes. The reduction of LDLR promotes plasmatic the rise of LDL levels and atherosclerosis development, and inhibitors of PCSK9 substantially decrease them and reduce cardiovascular risk [130].

Browning is probably a valuable tool for the primary prevention of atherosclerosis because it attenuates plaque growth. Nevertheless, the initial observations of its probable ineffectiveness in atherosclerotic plaque regression and stability suggest a lack of efficiency in advanced disease [125,126]. Compared to statins and PCSK9 inhibitors, induction of browning may not be a pillar treatment. However, it seems a good co-treatment for atheroprotection, particularly in obese patients, because of a conjoint weight-loss advantage. Recent data suggest that BAT activation via β3-adrenoreceptor agonists in mice combined with PCSK9 inhibitors enhances their atheroprotective efficacity, tending to reduce atherogenesis compared to PCSK9 inhibitors monotherapy [130]. Their combined action potentiates the decrease of total cholesterol and the increase of the hepatic clearance for the triglyceride-rich lipoprotein remnants, and improves plasma HDL levels [130].

Some restrictions may apply to the potential use of WAT browning as an anti-atherogenic treatment. First, the potential of beige adipocyte differentiation is not fully understood. Even if it seems attractive, especially in overweight people, the WAT trans-differentiation turnover may reach a limit and may be insufficient for a therapeutic benefit. Besides, the induction of beige adipocytes in subjects with an average body weight may be very low because of the small amount of WAT, suggesting a minor therapeutic potential in this context. Secondly, cold exposure is constraining. Five hours of exposure to an 18 °C temperature raises the energy expenditure by 7.5% [131]. Though, thermogenic AT turns 127 kcal into heat for a 70 kg person, suggesting that a loss of 0.5 kg requires a daily cold exposure during a month [131]. Furthermore, drugs that may induce browning, such as β3-adrenoreceptor agonists, have shown extremely low efficacity for now [15]. Finally, the beige and brown adipocytes preserve the body temperature via adaptive thermogenesis. Drug-induced activation of thermogenic AT may thus also impair adaptive thermogenesis, its primary function [132]. For now, no browning activator can replace chronic cold exposure in humans. Advancing in thermogenic AT research is needed to deepen our understanding of its close relation to atherosclerosis. Therapeutic target investigations need to be further conducted in order to uncover this exciting field.
